# CITRINO: phase 1 dose escalation study of anti-LAG-3 antibody encelimab alone or in combination with anti-PD-1 dostarlimab in patients with advanced/metastatic solid tumours

**DOI:** 10.1038/s44276-024-00118-x

**Published:** 2025-02-27

**Authors:** J. Randolph Hecht, Jean-Marie Michot, David Bajor, Amita Patnaik, Ki Y. Chung, Judy Wang, Gerald Falchook, James M. Cleary, Richard Kim, Anuradha Krishnamurthy, Omkar Marathe, Hagop Youssoufian, Catherine Ellis, Angela Waszak, Srimoyee Ghosh, Hailei Zhang, Kaitlin Yablonski, Shruti D. Shah, Ivan Diaz-Padilla, Susanna Ulahannan

**Affiliations:** 1 David Geffen School of Medicine, University of California Los Angeles (UCLA), Los Angeles, CA USA; 2 Département d’Innovation Thérapeutique et d’Essais Précoces, Institut de Cancérologie Gustave Roussy, Villejuif, France; 3 Department of Medicine, Case Western Reserve University, University Hospitals Cleveland Medical Center, Cleveland, OH USA; 4 START San Antonio, San Antonio, TX USA; 5 Department of Medicine, Prisma Health Cancer Institute, Greenville, SC USA; 6 Drug Development Unit, Florida Cancer Specialists/Sarah Cannon Research Institute, Sarasota, FL USA; 7 Drug Development Unit, Sarah Cannon Research Institute at HealthONE, Denver, CO USA; 8 Department of Medical Oncology, Dana-Farber Cancer Institute, Boston, MA USA; 9 Department of Medical Oncology, H. Lee Moffitt Cancer Center, Tampa, FL USA; 10 Department of Medicine, Roswell Park Comprehensive Cancer Center, Buffalo, NY USA; 11 The Oncology Institute of Hope and Innovation, Whittier, CA USA; 12 Oncology Clinical Development, GSK, Waltham, MA USA; 13 Oncology Clinical Development, GSK, Collegeville, PA USA; 14 Oncology Clinical Development, GSK, Zug, Switzerland; 15 Stephenson Cancer Center, University of Oklahoma, Oklahoma City, OK USA; 16 Sarah Cannon Research Institute, Nashville, TN USA

## Abstract

**Background:**

Dual programmed cell death protein (ligand)-1 (PD-[L]1) and lymphocyte-activation gene-3 (LAG-3) blockade has demonstrated improved anti-tumour response in some advanced solid tumours. CITRINO, a two-part, Phase 1 dose-escalation study, evaluated encelimab (TSR-033; novel anti-LAG-3) monotherapy and in combination in patients with advanced/metastatic solid tumours.

**Methods:**

Part 1 (P1) involved dose escalation (20–720 mg Q2W) of encelimab as monotherapy (P1A/B) and with dostarlimab (500 mg Q3W) in patients with previously treated advanced/metastatic solid tumours (P1C). P2 involved cohort expansion in patients with anti-PD-(L)1-naïve microsatellite stable advanced/metastatic colorectal cancer with recommended phase 2 dose (RP2D) of encelimab with dostarlimab as third/fourth-line therapy (P2A), or with dostarlimab, bevacizumab and mFOLFOX6/FOLFIRI as second-line therapy (P2B). Objectives included RP2D, safety/tolerability, efficacy, pharmacokinetics/pharmacodynamics, and exploratory biomarkers.

**Results:**

Maximum tolerated encelimab dose was not reached; 720 mg Q2W was used for P2 plus dostarlimab 1000 mg Q6W. One dose-limiting toxicity occurred (Grade 2 myasthenia gravis; P1A). No clinical responses were observed in P1; 1 (3%) and 4 (17%) patients achieved partial response in P2A and 2B, respectively.

**Conclusions:**

Encelimab has a manageable safety profile as a monotherapy and in tested combinations; however, anti-tumour activity was limited.

**Clinical trial registration:**

NCT03250832.

## Background

Immune checkpoint pathways can be co-opted by cancer cells to evade immune detection and destruction. Key proteins within the immune checkpoint family include programmed cell death protein-1 (PD-1), programmed death ligand-1 (PD-L1), cytotoxic T-lymphocyte associated protein 4 (CTLA-4), and lymphocyte-activation gene-3 (LAG-3) [1–4]. PD-(L)1 inhibitors have demonstrated considerable clinical activity and benefit across a spectrum of cancers, leading to approvals across multiple advanced solid tumour and haematologic malignancies and substantially shifting the treatment paradigm in recent years [5, 6]. However, not all patients respond to these treatments, and some relapse following a period of response [4, 7, 8]. As such, there is strong interest in combining PD-(L)1 inhibitors with other treatment agents, including LAG-3 inhibitors, chemotherapy, and biological agents, to improve clinical responses in advanced solid tumour types, including colorectal cancer (CRC) [4, 8, 9].

CRC is the third most common cancer globally, accounting for 10% of all new cancer cases, and remains the second most common cause of cancer death [10, 11]. Clinical efficacy of immune checkpoint inhibition has previously been demonstrated in mismatch repair deficient (dMMR)/microsatellite instability-high metastatic CRC in the first-line and later-line settings [12–14]. By contrast, immune checkpoint inhibitors have shown limited benefit in the more prevalent microsatellite-stable (MSS) CRC, which accounts for 85–97% of CRC cases depending on stage [15–17]. Current standard of care for MSS metastatic CRC in first- and second-line settings includes chemotherapy, such as FOLFOX (folinic acid, 5-fluorouracil, oxaliplatin) or FOLFIRI (folinic acid, fluorouracil, irinotecan) regimens, with or without anti-vascular endothelial growth factor/epidermal growth factor receptor (EGFR) treatment [18]. Treatment options in later lines include chemotherapy and targeted therapies [18]. However, not all patients respond to available therapies, and long-term benefit is modest and transient, highlighting the unmet need for novel treatment regimens in MSS metastatic CRC [19].

LAG-3 has become a target of interest in CRC in recent years, including interest in dual inhibition with PD-1. Expression of LAG-3 is upregulated on immune cells within CRC tumours, and high expression is associated with poor tumour differentiation, advanced stage, lymph node involvement, and invasion depth [20]. Further, a retrospective analysis demonstrated patients with LAG-3 positive colorectal tumours had significantly shorter survival that those with low LAG-3 tumour expression [20]. With regards to dual inhibition of LAG-3 and PD-1, early-stage clinical data have shown improved anti-tumour responses in patients with advanced solid tumours treated with anti-LAG-3 plus anti-PD-1 combinations [21]. More recently, the combination of nivolumab (anti-PD-1) and relatlimab (anti-LAG-3) has demonstrated strong anti-tumour activity in the neoadjuvant treatment of dMMR CRC, with a pathological response rate of 97% [22]. Additionally, early-stage clinical data suggest improved anti-tumour response in MSS CRC patients treated with favezelimab (anti-LAG-3) plus pembrolizumab (anti-PD-1), versus pembrolizumab monotherapy (objective response rate [ORR] 6.3% [*n* = 5/80] versus 0% [*n* = 0/20], respectively) [23]. Taken together, these findings support further investigation of dual LAG-3 and PD-1 inhibition in advanced solid tumours, including MSS CRC—an area of high unmet need.

Encelimab (TSR-033) is a novel selective humanised immunoglobulin G4 (IgG4) monoclonal antibody that binds and inhibits LAG-3 [24]. Preclinical studies in an in vivo non-small cell lung cancer model demonstrated improved anti-tumour activity when adding encelimab to dostarlimab, an anti-PD-1 antibody approved for the treatment of dMMR advanced solid tumours [24–26]. The combination resulted in significantly higher immune stimulation with increased total and proliferating intratumoural T cells, providing rationale for combining the two agents for improved anti-tumour activity [24]. Here we report results from the CITRINO (NCT03250832, registered June 22, 2017) study, a non-randomised, multi-centre, open-label, first-in human, Phase 1 dose-escalation study evaluating encelimab alone and in combination with dostarlimab in patients with previously treated advanced or metastatic solid tumours, which included cohort expansion evaluating the combination of encelimab and dostarlimab, with or without bevacizumab and mFOLFOX6 (modified FOLFOX) or FOLFIRI, in PD-(L)1-naïve advanced or metastatic MSS CRC.

## Methods

### Study design

The study was conducted in two parts, a dose-escalation stage (part 1) to identify the recommended Phase 2 dose (RP2D) of encelimab as monotherapy and in combination with dostarlimab in patients with previously treated advanced or metastatic solid tumours, and a cohort expansion stage (part 2) in patients with previously treated PD-(L)1-naïve MSS advanced or metastatic CRC (Supplementary Fig. 1). Part 1A consisted of dose escalation of encelimab monotherapy using a starting dose of 20 mg every 2 weeks (Q2W) with an initial 3 + 3 design, with planned escalation to 720 mg Q2W (Supplementary Fig. 1). In dose levels above 20 mg, 6 patients were initially enroled. Dose escalation (where dose-limiting toxicities [DLTs] were observed in less than one-third of patients) or expansion (where DLTs were observed in one-third of patients) was considered after all initial patients had completed the DLT observation period and were evaluable.

In part 1B (pharmacokinetic and pharmacodynamic characterisation), up to 6 additional patients per dose level were enroled at a dose level where DLTs were observed in less than one-third of patients, with planned escalation to 720 mg Q2W (Supplementary Fig. 1).

Part 1C consisted of dose escalation of encelimab in combination with dostarlimab 500 mg Q3W, where the starting dose of encelimab was one dose level below the highest dose at which less than one-third of patients experienced DLTs with single-agent encelimab (Supplementary Fig. 1).

In part 2, it was planned that patients with MSS CRC would receive encelimab at the RP2D in combination with dostarlimab 1000 mg Q6W (1000 mg Q6W was selected based on the similar pharmacokinetic and safety profiles to 500 mg Q3W [27], and for patient convenience) in third- or fourth-line treatment (part 2A, *n* ≈ 31), or as second-line treatment with bevacizumab (Q2W) plus mFOLFOX6 or FOLFIRI (Q2W; part 2 cohort B1 and cohort B2, respectively; both *n* = 6–12; Supplementary Fig. 1). In all parts of the study, treatment continued for up to 2 years or until disease progression, unacceptable toxicity, patient withdrawal, investigator decision, or death.

The study took place across five sites in the US and one site in France (part 1A/B and C), 10 sites in the US (part 2A), and eight sites in the US (part 2B). The study protocol was reviewed and approved by relevant ethics committees or institutional review boards at each site in accordance with International Conference on Harmonization Good Clinical Practice (ICH GCP) and applicable country-specific requirements. All patients provided written informed consent before participation in the study. This study was conducted in accordance with ICH GCP guidelines and the Declaration of Helsinki. Patients were not involved in the design, conduct or reporting of this study.

### Objectives

For part 1 (advanced or metastatic solid tumours) of the study, primary objectives were to define the RP2D and schedule of encelimab as a monotherapy and in combination with dostarlimab and evaluate the safety and tolerability of both regimens. For part 2A, the primary objective was to evaluate anti-tumour activity of encelimab plus dostarlimab in anti-PD-(L)1-naïve patients who had progressed after 2–3 lines of therapy. In part 2B, the primary objective was to evaluate the safety and tolerability of encelimab and dostarlimab in combination with bevacizumab and mFOLFOX6 (cohort B1) or FOLFIRI (cohort B2) in anti-PD-1-naïve patients following progression on first-line FOLFOX or FOLFIRI (or variants, with or without biologics), respectively.

Part 1 and 2 secondary objectives included characterisation of the pharmacokinetics and immunogenicity of all investigational regimens, and evaluation of ORR (part 1 and 2), duration of response (DoR; part 2), and disease control rate (DCR; part 2). Part 1 and 2 exploratory objectives included characterising the pharmacodynamic profile of encelimab and dostarlimab and identifying a biomarker-based patient population that would derive clinical benefit from treatment with encelimab in combination with dostarlimab.

### Patient population

#### Inclusion criteria

All patients were required to be ≥18 years of age, have an Eastern Cooperative Oncology Group performance status (ECOG PS) of 0 or 1, and have adequate haematologic and organ function. Key eligibility criteria for part 1 included patients with any histologically or cytologically confirmed advanced or metastatic solid tumour and progressive disease (PD) after treatment with available therapies or who were intolerant to treatment, determined based on patient records. In part 2, key eligibility criteria included histologically or cytologically confirmed CRC that was metastatic or not amenable to potentially curative resection, primary and/or metastatic tumour that was known to be MSS (as determined locally), and measurable disease per response evaluation criteria in solid tumours (RECIST) v1.1. For part 2A, eligible patients received 2–3 prior lines of therapy in an advanced or metastatic setting and progressed on standard therapies (or withdrawn due to unacceptable toxicity) including fluoropyrimidine, oxaliplatin, irinotecan, bevacizumab and/or another anti-angiogenic agent, and, if RAS-wild-type, an anti-EGFR agent, such as cetuximab or panitumumab. For part 2B, eligible patients received fewer than 2 prior systemic chemotherapy regimens (only 1 prior regimen for metastatic disease permitted) and have received first-line combination therapy consisting of bevacizumab or anti-EGFR antibodies with FOLFIRI (cohort B1) or FOLFOX (for cohort B2) and experienced radiographic progression.

#### Exclusion criteria

Key exclusion criteria for all parts of the study included patients with prior anti-LAG-3 treatment, known uncontrolled central nervous system metastases and/or carcinomatous meningitis, prior anti-cancer therapy within 21 days or less than 5 times the half-life of the most recent therapy prior to the first dose of study drug, and those who had not recovered from radiation- and chemotherapy-induced adverse events (AEs). For part 2, patients with prior anti-PD-(L)1 treatment were excluded.

### Interventions and assessments

#### Treatments

Encelimab and dostarlimab were administered via 30-min intravenous (IV) infusion. FOLFIRI included fluorouracil, irinotecan, and leucovorin; mFOLFOX6 included a modified schedule of oxaliplatin, fluorouracil, and leucovorin (dosage available in Supplementary Fig. 1). For combination regimens, encelimab was administered first, prior to dostarlimab; for part 2B bevacizumab and mFOLFOX6/FOLFIRI were administered 2 days before encelimab and dostarlimab.

#### Assessments

Safety assessments were conducted throughout the study and included physical examination, safety evaluation (including DLTs, serious adverse events [SAEs], treatment-emergent adverse events [TEAEs], and immune-related adverse events [irAEs]), vital signs, electrocardiograms, ECOG PS evaluation, and clinical laboratory assessments. The primary efficacy endpoint (part 2A) was ORR and was defined as the achievement of complete response (CR) or partial response (PR) per RECIST v1.1 and investigator assessment. Secondary efficacy endpoints included ORR (parts 1 and 2B), DoR (part 2), and DCR (part 2, defined as the proportion of patients achieving CR, PR, or stable disease [SD] for at least 12 weeks, per RECIST v1.1 and investigator assessment). Disease assessments (computed tomography [CT], magnetic resonance imaging, or positron emission tomography/CT) were performed Q6W for the first 3 assessments, Q9W until 1 year on treatment, and Q12W thereafter. Blood and tumour samples were collected for pharmacokinetic, pharmacodynamic, and biomarker analysis. Serum samples for pharmacokinetic determination via enzyme-linked immunosorbent assay were collected pre- and post-dose throughout the study, with regular sampling post-dose 1 and 6 for part 1 (+0.25, 0.5, 1.0, 1.5, 3, 24, 48, 96, 168, 336 [part 1B dose 1 and part 1C dose 1 and 6 only] h). Encelimab pharmacokinetic parameters were determined via standard non-compartmental methods using Phoenix WinNonlin version 8.2.2 based on actual sampling time. Pharmacokinetic parameters are presented as geometric mean (coefficient of variation) and median (range) where appropriate. Serum samples for immunogenicity analyses for part 1 were collected at the same time, and pre-dose and post-infusion for part 2; samples were analysed for the presence of anti-encelimab antibodies in a tiered testing strategy using a validated electrochemiluminescence immunoassay. Immunohistochemistry was used to measure PD-L1 (SP263 Ventana assay) and LAG-3 (clone 17B4) expression, determined centrally. PD-L1 positivity was defined by visually estimated combined positive score (vCPS; sum of PD-L1+ tumour cells and PD-L1+ immune cells divided by tumour area) of ≥1, and LAG-3 positivity was defined by a LAG-3 (LAG-3+ immune cells divided by tumour area) score of ≥1. Longitudinal blood samples were collected before and during encelimab treatment to analyse blood cells for LAG-3 receptor occupancy using a dual detection assay. Receptor occupancy was expressed as a ratio of T-cell-bound encelimab to total LAG-3 and normalised to baseline (pre-dose) for each patient. Assessment of circulating tumour DNA was optional and exploratory, and therefore not completed for all patients.

### Sample size

Approximately 132 patients were expected to be enroled across part 1. For part 2A, a null hypothesis of ORR 10% was tested against an alternative hypothesis of ORR 25%. A target sample size of 31 patients would attain a power of 82.4% (alpha level 0.1) and an attained type-1 error of 0.083. For part 2B, a null hypothesis of ORR 20% was tested against an alternative hypothesis of ORR 40%. A target sample size of 24 patients would attain a power of 80.8% (alpha level 0.1) and an attained type-1 error of 0.089.

### Statistical analyses

All descriptive statistical analyses were performed using SAS statistical software version 9.4. Categorial variables included calculated number and percent of each category; continuous variables included sample size, mean, median, standard deviation, first quartile, third quartile, and minimum/maximum values. Time-to-event analyses were performed with Kaplan-Meier methodology using 25th, 50th, and 75th percentiles and associated 2-sided 95% confidence intervals (CIs). AEs were coded according to Medical Dictionary for Regulatory Activities (MedDRA) v24.1 or later; severity was reported according to Common Terminology Criteria for Adverse Events v5. The safety population included all patients who received any amount of any study drug. The efficacy population included all participants who received any amount of encelimab. The pharmacokinetic, immunogenicity and receptor occupancy evaluable population included all patients with measurable samples who received any amount of encelimab.

## Results

### Patient population

Between August 8, 2017 (first patient first visit) and June 2, 2022 (last patient visit), the numbers of patients enroled and treated in part 1A/B, 1C, 2A, and 2B were 34, 18, 34, and 25, respectively (Supplementary Table 1). At data cut-off (June 2, 2022), all patients had discontinued treatment, with disease progression being the most common reason for discontinuation across treatment groups (Supplementary Table 1).

#### Patient demographics and baseline characteristics

Generally, patient demographics were similar across study parts (Table 1). There was a similar proportion of male and female patients across all parts of the study except part 2B, in which 83% of patients were male. Across all parts of the study, most patients were white (67–82%). Median age (range) of patients was 63 (22–84) years in part 1A/B, 60 (31–73) years in part 1C, 60 (27–77) years in part 2A, and 52 (36–76) years in part 2B. Most patients had ECOG PS of 1.

**Table 1:** Patient demographics and baseline characteristics for parts 1 and 2.

Characteristic	Part 1 A/B	Part 1 C	Part 2 A	Part 2B
	Encelimab monotherapy *N* = 34	Encelimab + dostar *N* = 18	Encelimab + dostar *N* = 34	Encelimab + dostar + bev + mFOLFOX/FOLFIRI *N* = 25
Sex, *n* (%)				
Female	18 (52.9)	10 (55.6)	18 (52.9)	4 (16.7)
Male	16 (47.1)	8 (44.4)	16 (47.1)	20 (83.3)
Race, *n* (%)				
White	25 (73.5)	12 (66.7)	28 (82.4)	17 (70.8)
Black	2 (5.9)	0	4 (11.8)	3 (12.5)
Asian	1 (2.9)	1 (5.6)	1 (2.9)	1 (4.2)
American Indian or Alaska Native	0	1 (5.6)	0	2 (8.3)
Other	6 (17.6)	4 (22.2)	1 (2.9)	1 (4.2)
Median age, years (range)	63.0 (22–84)	60.0 (31–73)	59.5 (27–77)	51.5 (36–76)
ECOG PS, *n* (%)				
0	12 (35.3)	6 (33.3)	14 (41.2)	12 (50.0)
1	22 (64.7)	12 (66.7)	20 (58.8)	12 (50.0)

*Bev* bevacizumab, *dostar* dostarlimab, *ECOG PS* Eastern Cooperative Oncology Group performance status, FOLFIRI folinic acid, fluorouracil, irinotecan; *mFOLFOX* modified folinic acid, 5-fluorouracil, oxaliplatin.

The most common primary tumour site in part 1A/B was prostate (*n* = 6, 18%) and in part 1C was head and neck (*n* = 3, 17%) and thymus (*n* = 3, 17%) (Supplementary Table 2). In parts 2A and B, all patients had colorectal adenocarcinoma (Supplementary Table 3). In part 2A, most patients had received 2 (*n* = 16, 47%) or 3 (*n* = 16, 47%) lines of prior treatment and in part 2B, 97% (*n* = 22) of patients had received 1 line of prior treatment, reflecting the study cohort inclusion criteria (Supplementary Table 3). Bevacizumab was the most commonly prescribed prior regimen for both part 2A (*n* = 30, 88%) and part 2B (*n* = 20, 83%). In part 2A, 38% (*n* = 13) of patients had LAG-3 positive tumours while in part 2B, 58% (*n* = 14) of patients had LAG-3 positive tumours (Supplementary Table 3). In part 2A, 12 patients (35%) had tumours that were PD-L1 positive, while in part 2B, 6 patients (25%) had tumours that were PD-L1 positive (Supplementary Table 3).

### Dose escalation and safety

One DLT was observed in the study; myasthenia gravis (Grade 2) occurred in 1 patient at a dose level of 80 mg encelimab monotherapy in part 1A. No treatment was administered for the event, and the patient recovered within 21 days. The maximum tolerated dose of encelimab was not reached in part 1A/B or part 1C, and dose escalation continued to a maximally administered dose of 720 mg encelimab in part 1A/B and 720 mg encelimab in combination with dostarlimab 500 mg in part 1C. This, in combination with pharmacodynamic data, suggested that increased doses would unlikely yield increased receptor occupancy, and resulted in the maximum administered dose of 720 mg encelimab being taken forward to part 2.

Overall, 33 patients (97%) experienced at least 1 TEAE in part 1A/B, 18 (100%) in part 1C, 32 (94%) in part 2A, and 25 (100%) in part 2B (Table 2). The most commonly reported TEAEs were arthralgia (*n* = 9, 27%) and decreased appetite (*n* = 7, 21%) in part 1A/B; fatigue (*n* = 8, 44%) in part 1C; fatigue (*n* = 10, 29%) and infusion-related reaction (*n* = 10, 29%) in part 2A; and fatigue (*n* = 13, 52%) and nausea (*n* = 13, 52%) in part 2B (Supplementary Table 4). Treatment-related TEAEs (TRAEs) were experienced by 14 patients (41%; Table 2) in part 1A/B, the most common being nausea (*n* = 3, 9%), and 10 patients (56%; Table 2) in part 1C, the most common being fatigue (*n* = 6, 33%). In part 2A, 26 patients (77%) experienced TRAEs, most commonly an infusion-related reaction (*n* = 10, 29%), and in part 2B, 20 (80%) experienced TRAEs, with fatigue (*n* = 9, 36%) most common. Grade ≥3 TRAEs were reported for no patients in part 1A/B and 3 patients (17%) in part 1C, 4 (12%) in part 2A, and 8 (32%) in part 2B (Table 2). Immune-related TEAEs (irTEAEs) were experienced by 5 patients (15%) in part 1A/B and 12 patients (67%) in part 1C. The most common irTEAE was arthralgia in both part 1A/B (*n* = 3, 9%) and 1C (*n* = 4, 22%; Supplementary Table 5). Grade 3 irTEAEs were experienced by 1 patient (3%) in part 1A/B (arthralgia) and 3 patients (17%) in part 1C (2 cases of arthralgia and 1 of rash maculo-papular). In part 2A, 16 patients (47.1%) experienced an irTEAE; the most common irTEAE was infusion-related reaction (*n* = 10, 29%; Supplementary Table 5). Two patients (6%) experienced Grade 3 irTEAEs, 1 case of alanine aminotransferase increase and 1 case of pneumonitis. In part 2B, 11 patients (44.0%) experienced an irTEAE, the most common being hypothyroidism and infusion-related reaction (both *n* = 5, 20%; Supplementary Table 5). Grade 3 irTEAEs were experienced by 5 patients (20%) in part 2B, 2 of whom experienced aspartate aminotransferase increase, 1 alanine aminotransferase increase, 1 colitis, and 1 encephalitis. The infusion-related reactions observed in part 2 were thought to be related to encelimab, as this event only occurred on encelimab infusion days. These events were non-serious and manageable. No Grade 4 or 5 irTEAEs were observed in any study parts.

**Table 2:** Summary of treatment-emergent adverse events for part 1 and 2.

Event, n (%)	Part 1A/B					Part 1C				Part 2A	Part 2B		
	Encelimab 20 mg *N* = 3	Encelimab 80 mg *N* = 10	Encelimab 240 mg *N* = 11	Encelimab 720 mg *N* = 10	Total *N* = 34	Encelimab 80 mg + dostar 500 mg *N* = 5	Encelimab 240 mg + dostar 500 mg *N* = 7	Encelimab 720 mg + dostar 500 mg *N* = 6	Total *N* = 18	Encelimab 720 mg + dostar 1000 mg *N* = 34	Encelimab 720 mg + dostar 1000 mg + bev + mFOLFOX6 *N* = 4	Encelimab 720 mg + dostar 1000 mg + bev + FOLFIRI *N* = 21	Total *N* = 25
Any TEAE	3 (100)	10 (100)	10 (90.9)	10 (100)	33 (97.1)	5 (100)	7 (100)	6 (100)	18 (100)	32 (94.1)	4 (100)	21 (100)	25 (100)
TRAEs	1 (33.3)	3 (30.0)	6 (54.5)	4 (40.0)	14 (41.2)	2 (40.0)	4 (57.1)	4 (66.7)	10 (55.6)	26 (76.5)	3 (75.0)	17 (81.0)	20 (80.0)
Grade ≥3 TEAEs	2 (66.7)	4 (40.0)	3 (27.3)	3 (30.0)	12 (35.3)	3 (60.0)	5 (71.4)	5 (83.3)	13 (72.2)	15 (44.1)	4 (100)	14 (66.7)	18 (72.0)
Grade ≥3 TRAEs	0	0	0	0	0	1 (20.0)	1 (14.3)	1 (16.7)	3 (16.7)	4 (11.8)	1 (25.0)	7 (33.3)	8 (32.0)
Treatment-emergent SAEs	1 (33.3)	5 (50.0)	2 (18.2)	1 (10.0)	9 (26.5)	1 (20.0)	3 (42.9)	4 (66.7)	8 (44.4)	7 (20.6)	4 (100)	7 (33.3)	11 (44.0)
Treatment-related SAEs	0	1 (10.0)	0	0	1 (2.9)	0	0	1 (16.7)	1 (5.6)	1 (2.9)	0	4 (19.0)	4 (16.0)
TEAEs leading to withdrawal of study treatment	0	3 (30.0)	1 (9.1)	1 (10.0)	5 (14.7)	0	0	0	0	1 (2.9)	0	5 (23.8)	5 (20.0)
TEAEs leading to death	0	0	0	0	0	0	1 (14.3)	0	1 (5.6)	0	0	1 (4.8)	1 (4.0)
Treatment-related TEAEs leading to death	0	0	0	0	0	0	0	0	0	0	0	0	0

Patient could be included in >1 event category.

*Bev* bevacizumab, *dostar* dostarlimab, *FOLFIRI* folinic acid, fluorouracil, irinotecan, *mFOLFOX* modified folinic acid, 5-fluorouracil, oxaliplatin, *SAE* serious adverse event, *TEAE* treatment-emergent adverse event, *TRAE* treatment-related TEAE.

### Anti-tumour activity

No clinical responses were observed in part 1 of the study; 9 patients (27%) in part 1A/B had the best overall response of SD and 20 (59%) of PD. In part 1C, 4 patients (22%) had the best overall response of SD and 12 (67%) of PD.

**Fig. 1:**
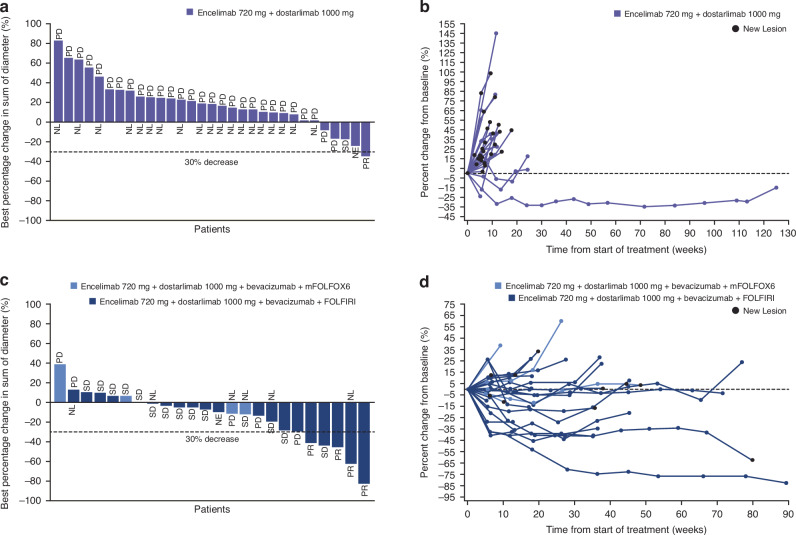
Anti-tumour activity in parts 2A and 2B. Best percentage tumour size change (**a**) and percentage change from baseline (**b**) for part 2A; encelimab plus dostarlimab. Best percentage tumour size change (**c**) and percentage change from baseline (**d**) for part 2B; encelimab plus dostarlimab plus bevacizumab plus mFOLFOX/FOLFIRI. FOLFIRI folinic acid, fluorouracil, irinotecan, mFOLFOX modified folinic acid, 5-fluorouracil, oxaliplatin, NE not evaluable, NL new lesion, PD progressive disease, PR partial response, SD stable disease.

No CRs were observed in part 2 of the study (Table 3, Fig. 1). One patient (3%) with lung and adrenal gland metastases in part 2A achieved a PR (Table 3, Fig. 1). In part 2B four patients (17%) achieved a PR; two with liver metastases, one with lung metastases and peritoneum mesenteric mass, and one with lung and liver metastases (Table 3, Fig. 1). Those patients who achieved PR demonstrated durable responses (Fig. 1), with an estimated median DoR (95% CI) of 23.3 (not evaluable [NE]–NE) months in part 2A and 14.1 (7.0–NE) months in part 2B. The DCR in part 2A and 2B was 3 (9%) and 18 (75%), respectively (Table 3).

**Table 3:** Tumour response summary per RECIST v1.1 for part 2.

Response measure	Part 2A	Part 2B		
	Encelimab 720 mg + dostar 1000 mg *N* = 34	Encelimab 720 mg + dostar 1000 mg + bev + mFOLFOX6 *N* = 4	Encelimab 720 mg + dostar 1000 mg + bev + FOLFIRI *N* = 20	Total *N* = 24
**Best overall response,** ***n*** **(%)**				
CR	0	0	0	0
PR	1 (2.9)	0	4 (20.0)	4 (16.7)
SD	2 (5.9)	2 (50.0)	12 (60.0)	14 (58.3)
PD	28 (82.4)	2 (50.0)	3 (15.0)	5 (20.8)
Not evaluable	1 (2.9)	0	1 (5.0)	1 (4.2)
Not done	2 (5.9)	0	0	0
Confirmed ORR				
*n* (%)	1 (2.9)	0	4 (20.0)	4 (16.7)
95% CI^a^	(0.1–15.3)	(0.0–60.2)	(5.7–43.7)	(4.7–37.4)
Response ongoing,^b^ *n* (%)	0	0	2 (50.0)	2 (50.0)
Disease control rate				
*n* (%)	3 (8.8)	2 (50.0)	16 (80.0)	18 (75.0)
95% CI^a^	(1.9–23.7)	(6.8–93.2)	(56.3–94.3)	(53.3–90.2)

*Bev* bevacizumab, *CI* confidence interval, *CR* complete response, *dostar* dostarlimab, *FOLFIRI* folinic acid, fluorouracil, irinotecan, *mFOLFOX* modified folinic acid, 5-fluorouracil, oxaliplatin, *ORR* objective response rate, *PD* progressive disease, *PR* partial response, *RECIST* response evaluation criteria in solid tumours, *SD* stable disease.

^a^Exact 2-sided 95% CI for the binomial proportion.

^b^all responders who had not died or progressed at data cut-off (including clinical progression), denominator for percentage is number of responders.

Best overall response by biomarker status was evaluated as exploratory analyses (Supplementary Table 6). Among the 22 patients in part 2A with available PD-L1 status, 12 (55%) were PD-L1 positive, among whom 1 (8%) achieved a PR. No patients with a negative PD-L1 status demonstrated response. LAG-3 status was available for 18 patients in part 2A, 13 (72%) of whom were LAG-3 positive. Of the LAG-3 positive patients, 1 (8%) achieved a PR. No patients with negative LAG-3 status demonstrated response. In part 2B, 6 patients (38%) were PD-L1 positive, none of whom demonstrated a response; 10 patients (38%) were PD-L1 negative, 2 (20%) of whom achieved PR. Among the 14 (93%) LAG-3 positive patients in part 2B, 2 (14%) achieved PR. No patients with negative LAG-3 status demonstrated response.

One patient receiving 720 mg encelimab plus 1000 mg dostarlimab plus bevacizumab plus FOLFIRI (part 2B) achieved a PR sustained for 20 months. The patient was diagnosed with stage IV rectal adenocarcinoma approximately 3 years prior to initiation of study treatment. Biomarker and mutational status were not evaluable due to insufficient tissue. Prior treatments included FOLFOX plus panitumumab for 8 cycles followed by 5-fluorouracil plus panitumumab, which was switched to bevacizumab plus capecitabine due to toxicity; a best response of SD was demonstrated. The patient underwent a colostomy in the year prior to study treatment initiation to manage an obstructing and bleeding tumour; the primary tumour was not removed. The patient was then diagnosed with PD with enlarging primary tumour and liver metastasis. Upon initiating study treatment, a PR was achieved within 1 month and sustained throughout study treatment duration (21 months). The patient had a single baseline target lesion in the liver measuring 51 mm, which was reduced to 32 mm (−37.5%) at the first tumour assessment (Week 6), 12 mm (−76%) after 1 year of study treatment (Week 78), 9 mm (−82.3%) 3 weeks post-study treatment discontinuation (Week 90; Supplementary Fig. 2). The patient also had non-target liver lesions and primary tumour in place. Treatment was discontinued at the physician’s discretion due to surgery, and a pathological CR was confirmed post-surgery. At data cut-off (30 months after study treatment initiation) the patient was alive with no evaluable disease and negative circulating tumour DNA.

### Pharmacokinetics

**Fig. 2:**
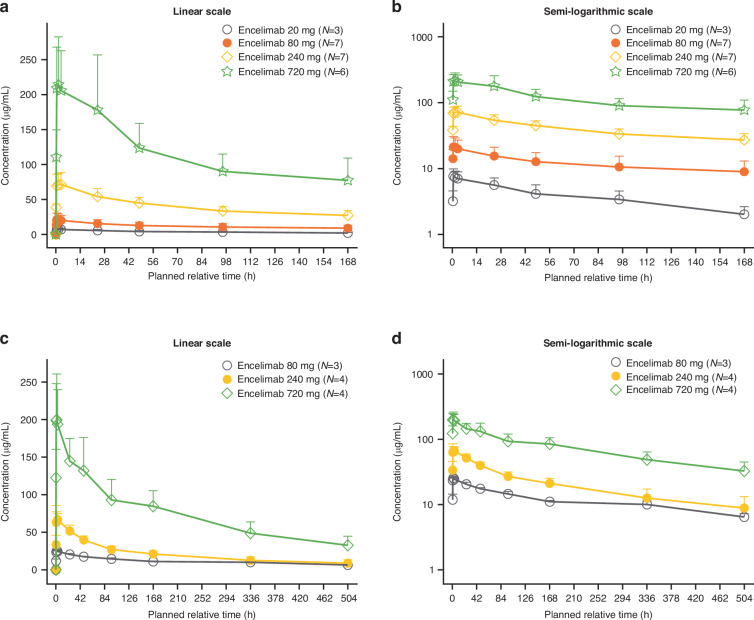
Mean serum concentration-time profile of encelimab dose 1 in parts 1A and 1B. Mean serum concentration time profile plotted on a linear scale (**a**, **c**) and semi-logarithmic scale (**b**, **d**) for encelimab dose 1 in part 1A (**a**, **b**) and part 1B (**c**, **d**); both encelimab monotherapy. Data points show mean ± standard deviation.

The mean concentration-time profile of encelimab in part 1A and 1B increased in a dose-proportional manner for the evaluated dose range (Fig. 2). When administered in combination with dostarlimab (part 1C), mean concentration-time profiles for encelimab were similar to monotherapy (Supplementary Fig. 3). In part 1A/B (encelimab monotherapy), no dose-related trend was observed for clearance (CL), volume of distribution at steady state (Vss), or terminal half-life (Table 4). The mean maximum concentration (*C*_max_) for encelimab increased in an approximately dose-proportional manner, and the median time at *C*_max_ (*T*_max_) was ~0.5–1.5 h post-dose across dose levels. The mean CL of encelimab monotherapy ranged from 0.02 L/h to 0.03 L/h, and mean Vss ranged from 3.48 L to 5.26 L across part 1A/B (Table 4). When administered in combination with dostarlimab (part 1C), pharmacokinetic parameters were similar to those observed with monotherapy (Table 4).

**Table 4:** Summary of encelimab pharmacokinetics at first dose for part 1.

Pharmacokinetic parameter	Part 1 A/B				Part 1 C		
	Encelimab 20 mg *N* = 3	Encelimab 80 mg *N* = 10	Encelimab 240 mg *N* = 11	Encelimab 720 mg *N* = 10	Encelimab 80 mg + dostar 500 mg *N* = 5	Encelimab 240 mg + dostar 500 mg N = 7	Encelimab 720 mg + dostar 500 mg N = 6
*C*_max_ (μg/mL), GM (%CV)	7.49 (30.8) (*N* = 3)	22.81 (29.9) *N* = 10	74.13 (21.4) *N* = 10	217.1 (34.8) *N* = 10	23.04 (29.09) *N* = 5	73.5 (13.8) *N* = 7	312.8 (79.4) *N* = 6
*T*_max_ (h), median (range)	0.57 (0.52–1.55) (*N* = 3)	1.04 (0.27–3.05) *N* = 10	1.49 (0.47–3.02) *N* = 10	1.02 (0.5–24) *N* = 10	1.07 (1–1.57) *N* = 5	0.52 (0.47–3) *N* = 7	1.49 (0.5–3.03) *N* = 6
AUC (0–inf) (h*μg/mL), GM (%CV)	858.37 (39.33) (*N* = 2)	2783.44 (112.71) *N* = 3	12517.04 (37.28) *N* = 5	44300.89 (32.48) *N* = 5	3201.67 (35.59) *N* = 5	12336.28 (26.18) *N* = 4	52144.26 (15.62) *N* = 3
t1/2 (h), GM (%CV)	119.79 (19.14) (*N* = 3)	240.54 (72.79) *N* = 8	198.88 (39.33) *N* = 10	214.25 (32.64) *N* = 8	140.46 (18.34) *N* = 5	241.03 (25.78) *N* = 6	226.50 (26.71) *N* = 5
Vss (L), GM (%CV)	3.48 (36.61) (*N* = 3)	5.06 (33.19) *N* = 8	4.83 (21.6) *N* = 10	5.26 (31.24) *N* = 8	4.82 (36.86) *N* = 5	5.4 (17.33) *N* = 6	4.38 (32.8) *N* = 5
CL (L/h), GM (%CV)	0.02 (39.3) (*N* = 2)	0.03 (112.7) *N* = 3	0.02 (37.3) *N* = 5	0.02 (32.5) *N* = 5	0.03 (35.59) *N* = 5	0.02 (26.18) *N* = 4	0.01 (15.62) *N* = 3

*AUC* area under the concentration-time curve, *CL* clearance, *C*_*max*_ maximum concentration, CV coefficient of variation, dostar dostarlimab, GM geometric mean, h hours, inf infinity, *t*_*1/2*_, terminal half-life, *T*_*max*_ time at maximum concentration, *Vss* volume of distribution at steady state.

### Immunogenicity

Overall, 5% of patients (6/110) developed treatment-emergent anti-encelimab antibodies, with 7 instances recorded and with no overt impact on pharmacokinetics, safety, and efficacy. One instance was detected in part 1A/B (encelimab 20 mg, Dose 6, Day 1), 2 instances in part 1C (encelimab 80 mg plus dostarlimab 500 mg; 1 on Dose 2, Day 1, and 1 at end of treatment visit), 3 instances in part 2A (Dose 2, Day 1 [*n* = 2] and 90-day safety follow-up [*n* = 1]), and 1 instance in part 2B (encelimab 720 mg plus dostarlimab 1000 mg plus bevacizumab plus FOLFIRI, Dose 14, Day 3).

### Receptor occupancy

LAG-3 receptor occupancy was evaluated as an exploratory analysis. In part 1C, receptor occupancy was dose-proportional (Supplementary Fig. 4A, B). In patients treated with 80 mg encelimab, receptor occupancy was highest at Cycle 1 Day 5 and then decreased over the treatment period. By comparison, receptor occupancy in patients treated with 240 mg and 720 mg of encelimab plateaued at Cycle 1 Day 5 and was maintained over the treatment period, supporting the selection of 720 mg encelimab for part 2. In part 2A and 2B, receptor occupancy was variable across the patient cohort but was generally increased at Cycle 2 Day 1 and maintained over the treatment period (Supplementary Fig. 4C, D).

## Discussion

This first-in-human, multi-centre, open-label, Phase 1 dose-escalation study (CITRINO, NCT03250832) of encelimab, a novel LAG-3 inhibitor, evaluated encelimab alone and in combination with dostarlimab in patients with previously treated advanced or metastatic solid tumours, including cohort expansion evaluating the combination with or without bevacizumab and mFOLFOX6 or FOLFIRI in PD-(L)1-naïve advanced or metastatic MSS CRC.

The maximum tolerated dose of encelimab was not reached, consistent with other Phase 1 trials of LAG-3 inhibitors [21, 28, 29]; the maximum administered dose (720 mg) was used for cohort expansion (part 2) based on receptor occupancy data. Encelimab demonstrated a manageable safety profile as both monotherapy and in combination with dostarlimab with or without chemotherapy and bevacizumab, with only 1 DLT of Grade 2 myasthenia gravis reported in part 1 of the study. The safety profiles support a lack of synergistic toxicity of encelimab and dostarlimab. The manageable safety profile and reported TEAEs are in line with data from other Phase 1/2 trials evaluating anti-LAG-3 antibodies alone and in combination with anti-PD-1 antibodies in advanced/metastatic solid or haematological malignancies [21, 28, 29].

The plasma concentration of encelimab increased in a dose-proportional manner for the evaluated dose range, and the pharmacokinetic profiles of encelimab as a monotherapy and in combination with dostarlimab were comparable. Although treatment-emergent anti-encelimab antibodies were detected in a minority of patients, this had no overt impact on encelimab pharmacokinetics, safety, or efficacy. Encelimab LAG-3 receptor occupancy increased dose-proportionally in part 1 C and was generally increased and stable during treatment in part 2A and 2B, supporting the encelimab dosing regimen utilised in part 2.

Overall, clinical activity of encelimab as both monotherapy and combination therapy was limited across all primary and key secondary efficacy endpoints; however, the limited number of patients who did respond demonstrated durable responses. The deepest response was achieved by a patient with MSS CRC who had received 1 prior line of therapy before entering the study. The patient achieved a PR, which was sustained for 20 months and accompanied by a−76% tumour size reduction in part 2B (encelimab 720 mg plus dostarlimab 1000 mg plus bevacizumab plus FOLFIRI). At data cut-off the patient was alive with no evaluable disease and negative circulating tumour DNA. However, the overall limited clinical activity is consistent with observations from other early phase studies. In a Phase 1 study (MK-4280-001; NCT02720068) evaluating anti-LAG-3 IgG4 monoclonal antibody favezelimab in MSS CRC, no patients achieved clinical response with monotherapy treatment, and 6.3% achieved an objective response when administered in combination with an anti-PD-1 [23]. The median DoR for the combination was shorter than reported in CITRINO, at 10.6 months [23]. In an all-comers study of ieramilimab (anti-LAG-3 IgG4 monoclonal antibody) in advanced solid tumours, similar outcomes were reported, with no responses observed in the monotherapy arm and an ORR of 10.7% when combined with an anti-PD-1 [21]. In a Phase 1 study investigating tebotelimab, an IgG4 antibody that binds and inhibits LAG-3 and PD-1 concomitantly or independently, PR was achieved in 6.8% of patients with advanced solid tumours, with best response of SD for the 2 evaluable patients with CRC [29].

In this study, no clear association between clinical response and LAG-3 or PD-L1 biomarker status was observed based on exploratory analyses. Data from the Phase 1 MK-4280-001 study showed numerically higher response rates in patients with PD-L1 positive MSS CRC (CR in 2.8% and PR in 8.3% of patients) compared with those with negative PD-L1 status (no CRs, and PR in 2.9% of patients); response by LAG-3 status was not reported [23]. In a Phase 1 study of tebotelimab, response was found to be significantly correlated with increased LAG-3 expression in patients with advanced solid tumours; however, by contrast, no statistically significant correlation between PD-1 expression and response was observed. In the all-comers study of ieramilimab plus anti-PD-L1, neither PD-L1 nor LAG-3 were predictive of response in patients with advanced solid tumours [21]. Taken together, results from these trials support the need for further evaluation of LAG-3 and PD-L1 as predictive biomarkers. Further studies evaluating dual LAG-3 and PD-L1 inhibition are ongoing, including a Phase 3 randomised study investigating anti-LAG-3 plus anti-PD-L1 in metastatic CRC, which may provide further clarity on biomarker-based patient selection [30–32].

While the previously discussed studies report limited anti-tumour activity in advanced solid tumour or CRC cohorts, a study (RELATIVITY-047) evaluating relatlimab (IgG4 LAG-3-blocking antibody) in untreated advanced melanoma reported a significantly longer progression-free survival of 10.1 months when combined with anti-PD-1 nivolumab compared with 4.6 months for nivolumab alone [33]. However, RELATIVITY-047 was a study of untreated patients who were LAG-3 and PD-L1 positive [33]. By comparison, most patients enroled in part 2 of the CITRINO study had received at least 1 prior line of therapy and were not selected based on biomarker status. Of note, although all patients were LAG-3 and PD-L1 positive (defined as a combined positive score of greater than 1), RELATIVITY-047 found no correlation between LAG-3 or PD-L1 expression and treatment benefit [33].

A key limitation of CITRINO was the relatively unselected patient population. First, patients were not selected based on biomarker status. Analyses of response by PD-L1 and LAG-3 within the MSS population in part 2 of the study were exploratory and limited in sample size, and validation of these biomarkers in a larger population of patients with CRC would be valuable, in addition to evaluation of other biomarkers of interest such as tumour mutational burden and MSS status. Additionally, patient subanalyses by the presence of liver metastases and prior treatment for liver metastases were not performed. Liver metastases are known to significantly shorten survival in patients with CRC, and this therefore could have contributed to the lack of response observed [34]. Further, prior preclinical and clinical studies have shown liver metastases to be a negative predictor of immunotherapy response; however, literature here is mixed, with some suggestions of improved response with liver metastases [35–39].

The Phase 1 CITRINO study demonstrated encelimab has a manageable safety profile, as monotherapy and in combination with dostarlimab, for patients with previously treated advanced or metastatic solid tumours. Encelimab in combination with dostarlimab, bevacizumab, and mFOLFOX6 or FOLFIRI also demonstrated a manageable safety profile for patients with PD-(L)1-naïve advanced or metastatic MSS CRC. Encelimab pharmacokinetics were well characterised across dose levels. The pharmacokinetics, immunogenicity, and receptor occupancy parameters of encelimab were consistent across study treatment regimens and suggest no impact of dostarlimab addition. Overall, there was a lack of compelling evidence of clinical activity in PD-(L)1-naïve advanced or metastatic MSS CRC, supporting the need to further evaluate biomarkers predictive of response to anti-LAG-3 therapies and novel immunotherapies to improve outcomes in this patient population with high medical need.

## Supplementary information

Supplementary Material

## Data Availability

Please refer to GSK weblink to access GSK’s data sharing policies and as applicable seek anonymised subject level data via the link https://www.gsk-studyregister.com/en/.
