# Supplementary material for: CITRINO: phase 1 dose escalation study of anti-LAG-3 antibody encelimab alone or in combination with anti-PD-1 dostarlimab in patients with advanced/metastatic solid tumours
Source: BJC Rep. 2025 Feb 27;3:10. doi: 10.1038/s44276-024-00118-x (PMC11868598; doi:10.1038/s44276-024-00118-x)
Supplement: Supplementary file 1 — Supplementary Material [file 44276_2024_118_MOESM1_ESM.docx]

# SUPPLEMENTARY MATERIAL

**Supplementary Table 1.** Patient disposition (safety population) for part 1 and 2

| **Variable**  Reason, n (%) | **Part 1A/B**  N=34 | | | | **Part 1C** N=18 | | | **Part 2A** N=34 | **Part 2B** N=25 | |
| --- | --- | --- | --- | --- | --- | --- | --- | --- | --- | --- |
|  | **Encelimab**  **20 mg (N=3)** | **Encelimab**  **80 mg (N=10)** | **Encelimab 240 mg (N=11)** | **Encelimab 720 mg (N=10)** | **Encelimab**  **80 mg + dostar**  **500 mg (N=5)** | **Encelimab 240 mg + dostar**  **500 mg (N=7)** | **Encelimab 720 mg + dostar**  **500 mg (N=6)** | **Encelimab 720 mg + dostar 1000 mg (N=34)** | **Encelimab 720 mg + dostar**  **1000 mg + bev + mFOLFOX6 (N=4)** | **Encelimab 720 mg + dostar**  **1000 mg + bev + FOLFIRI (N=21)** |
| **Discontinued treatment** | **3 (100)** | **10 (100)** | **11 (100)** | **10 (100)** | **5 (100)** | **7 (100)** | **6 (100)** | **34 (100)** | **4 (100)** | **21 (100)** |
| AE | 0 | 1 (10) | 0 | 0 | 0 | 0 | 0 | 1 (2.9) | 0 | 2 (9.5) |
| Disease progression* | 3 (100) | 7 (70.0) | 10 (90.9) | 7 (70.0) | 5 (100) | 7 (100) | 5 (83.3) | 29 (85.3) | 3 (75.0) | 10 (47.6) |
| Withdrawal by patient | 0 | 1 (10.0) | 1 (9.1) | 2 (20.0) | 0 | 0 | 1 (16.7) | 3 (8.8) | 1 (25.0) | 3 (14.3) |
| Death | 0 | 0 | 0 | 0 | 0 | 0 | 0 | 0 | 0 | 2 (9.5) |
| Other | 0 | 1 (10) | 0 | 1 (10) | 0 | 0 | 0 | 1 (2.9) | 0 | 4 (19.0) |
| **Discontinued study** | **3 (100)** | **10 (100)** | **11 (100)** | **10 (100)** | **5 (100)** | **7 (100)** | **6 (100)** | **34 (100)** | **4 (100)** | **21 (100)** |
| Withdrawal of consent | 2 (66.7) | 4 (40.0) | 5 (45.5) | 5 (50.0) | 2 (40.0) | 1 (14.3) | 3 (50.0) | 8 (23.5) | 2 (50.0) | 7 (33.3) |
| Lost to follow-up | 0 | 0 | 0 | 0 | 0 | 0 | 0 | 3 (8.8) | 0 | 0 |
| Death | 1 (33.3) | 6 (60.0) | 5 (45.5) | 5 (50.0) | 3 (60.0) | 6 (85.7) | 3 (50.0) | 18 (52.9) | 1 (25.0) | 7 (33.3) |
| Sponsor decision | 0 | 0 | 1 (9.1) | 0 | 0 | 0 | 0 | 3 (8.8) | 1 (25.0) | 6 (28.6) |
| Other | 0 | 0 | 0 | 0 | 0 | 0 | 0 | 2 (5.9) | 0 | 1 (4.8) |

*Or based on clinical criteria by investigator.

AE, adverse event; bev, bevacizumab; dostar, dostarlimab; FOLFIRI, folinic acid, fluorouracil, irinotecan; mFOLFOX, modified folinic acid, 5-fluorouracil, oxaliplatin.

**Supplementary Table 2.** Additional patient baseline characteristics for part 1

| **Characteristic, n (%)** | **Part 1A/B**  **Encelimab monotherapy**  N=34 | **Part 1C**  **Encelimab + dostar** N=18 |
| --- | --- | --- |
| **Tumour site** | | |
| Bladder | 1 (2.9) | 1 (5.6) |
| Breast | 0 | 1 (5.6) |
| Bone | 2 (5.9) | 0 |
| Cervical | 0 | 1 (5.6) |
| Cholangiocarcinoma | 0 | 1 (5.6) |
| Colon | 4 (11.8) | 2 (11.1) |
| Endometrial | 4 (11.8) | 1 (5.6) |
| Gallbladder | 0 | 1 (5.6) |
| Head and neck | 1 (2.9) | 3 (16.7) |
| Kidney | 3 (8.8) | 0 |
| Left thigh | 0 | 1 (5.6) |
| Lung | 3 (8.8) | 0 |
| Maxillary bone | 1 (2.9) | 0 |
| Myxoid/round cell liposarcoma | 1 (2.9) | 0 |
| Nasopharyngeal | 0 | 1 (5.6) |
| Ovarian | 1 (2.9) | 0 |
| Pancreas | 1 (2.9) | 1 (5.6) |
| Periampullary | 1 (2.9) | 0 |
| Prostate | 6 (17.6) | 0 |
| Rectum | 1 (2.9) | 0 |
| Right fallopian tube | 1 (2.9) | 0 |
| Skin | 0 | 1 (5.6) |
| Thymic | 0 | 3 (16.7) |
| Tongue | 1 (2.9) | 0 |
| Urothelial | 1 (2.9) | 0 |
| Unknown | 1 (2.9) | 0 |
| **Known genetic abnormality in tumour** | | |
| *KRAS* | 5 (14.7) | 0 |
| *PI3K* | 0 | 1 (5.6) |
| *PTEN* | 1 (2.9) | 0 |
| *APC* | 1 (2.9) | 0 |
| *TP53* | 1 (2.9) | 0 |
| Other | 8 (23.5) | 6 (33.3) |
| Missing | 18 (52.9) | 11 (61.1) |

APC, APC regulator of WNT signalling pathway; dostar, dostarlimab; KRAS, KRAS proto-oncogene, GTPase; PI3K, phosphatidylinositol-4,5-bisphosphate 3-kinase catalytic subunit alpha; PTEN, phosphatase and tensin homolog; TP53, tumour protein P53.

**Supplementary Table 3.** Additional patient baseline characteristics for part 2

| **Characteristic, n (%)** | **Part 2A**  **Encelimab + dostar**  N=34 | **Part 2B**  **Encelimab + dostar + bev + mFOLFOX/FOLFIRI**  N=24 |
| --- | --- | --- |
| **Histology at diagnosis** | | |
| Adenocarcinoma | 34 (100) | 24 (100) |
| **Number of lines of prior treatments*** | | |
| 0 | 0 | 1 (4.2) |
| 1 | 2 (5.9) | 22 (97.1) |
| 2 | 16 (47.1) | 1 (4.2) |
| 3 | 16 (47.1) | 0 |
| **Main prior treatments received*** | | |
| Bevacizumab | 30 (88.2) | 20 (83.3) |
| Capecitabine | 10 (29.4) | 4 (16.7) |
| Oxaliplatin | 4 (11.8) | 4 (16.7) |
| Irinotecan | 4 (11.8) | 0 |
| Fluorouracil | 3 (8.8) | 6 (25.0) |
| Multiple^†^ | 33 (97.1) | 20 (83.3) |
| Other | 6 (17.6) | 2 (8.3) |
| **Baseline biomarker data** | | |
| LAG-3 | | |
| Positive | 13 (38.2) | 14 (58.3) |
| Negative | 5 (14.7) | 1 (4.2) |
| Missing | 16 (47.1) | 9 (37.5) |
| PD-L1 | | |
| Positive | 12 (35.3) | 6 (25.0) |
| Negative | 10 (29.4) | 10 (41.7) |
| Missing | 12 (35.3) | 8 (33.3) |

*Prior treatments include prior anti-cancer treatment where either extent of disease is metastatic and reason is palliative, or where extent of disease is locoregional, reason is neoadjuvant or adjuvant, and last prior anti-cancer treatment stop date is within 1 year of date of enrolment. ^†^Patients who received multiple treatments were counted in >1 listed treatment.

Bev, bevacizumab; dostar, dostarlimab; FOLFIRI, folinic acid, fluorouracil, irinotecan; LAG-3, lymphocyte activation gene-3; mFOLFOX, modified folinic acid, 5-fluorouracil, oxaliplatin; PD-L1, programmed death ligand-1.

**Supplementary Table 4.** Treatment-emergent adverse events

| **Preferred term, n (%)** | **Part 1A/B**  N=34 | | | | | **Part 1C**  N=18 | | | | **Part 2A** N=34 | **Part 2B** N=25 | | |
| --- | --- | --- | --- | --- | --- | --- | --- | --- | --- | --- | --- | --- | --- |
|  | **Encelimab 20 mg  (N=3)** | **Encelimab 80 mg   (N=10)** | **Encelimab 240 mg  (N=11)** | **Encelimab 720 mg  (N=10)** | **Total  (N=34)** | **Encelimab 80 mg + dostar**  **500 mg**  **(N=5)** | **Encelimab 240 mg + dostar**  **500 mg**  **(N=7)** | **Encelimab 720 mg + dostar**  **500 mg**  **(N=6)** | **Total (N=18)** | **Encelimab 720 mg + dostar 1000 mg (N=34)** | **Encelimab 720 mg + dostar 1000 mg + bev + mFOLFOX6 (N=4)** | **Encelimab 720 mg + dostar 1000 mg + bev + FOLFIRI (N=21)** | **Total (N=25)** |
| Abdominal pain | 0 | 1 (10.0) | 3 (27.3) | 1 (10.0) | 5 (14.7) | 0 | 0 | 0 | 0 | 0 | 1 (25.0) | 6 (28.6) | 7 (28.0) |
| Anemia | 0 | 0 | 0 | 0 | 0 | 0 | 0 | 0 | 0 | 7 (20.6) | 0 | 0 | 0 |
| Arthralgia | 2 (66.7) | 1 (10.0) | 3 (27.3) | 3 (30.0) | 9 (26.5) | 1 (20.0) | 2 (28.6) | 2 (33.3) | 5 (27.8) | 0 | 0 | 0 | 0 |
| Back pain | 0 | 1 (10.0) | 3 (27.3) | 2 (20.0) | 6 (17.6) | 2 (40.0) | 1 (14.3) | 2 (33.3) | 5 (27.8) | 0 | 0 | 0 | 0 |
| Constipation | 1 (33.3) | 1 (10.0) | 2 (18.2) | 2 (20.0) | 6 (17.6) | 0 | 0 | 0 | 0 | 7 (20.6) | 0 | 0 | 0 |
| Cough | 1 (33.3) | 0 | 3 (27.3) | 0 | 4 (11.8) | 0 | 0 | 0 | 0 | 0 | 0 | 0 | 0 |
| Decreased appetite | 0 | 4 (40.0) | 1 (9.1) | 2 (20.0) | 7 (20.6) | 1 (20.0) | 1 (14.3) | 4 (66.7) | 6 (33.3) | 6 (17.6) | 1 (25.0) | 4 (19.0) | 5 (20.0) |
| Diarrhea | 0 | 0 | 0 | 0 | 0 | 0 | 0 | 0 | 0 | 0 | 3 (75.0) | 9 (42.9) | 12 (48.0) |
| Dizziness | 0 | 0 | 0 | 0 | 0 | 0 | 0 | 0 | 0 | 0 | 2 (50.0) | 5 (23.8) | 7 (28.0) |
| Dry mouth | 1 (33.3) | 1 (10.0) | 1 (9.1) | 1 (10.0) | 4 (11.8) | 0 | 0 | 0 | 0 | 0 | 0 | 0 | 0 |
| Dyspnea | 1 (33.3) | 1 (10.0) | 0 | 2 (20.0) | 4 (11.8) | 2 (40.0) | 2 (28.6) | 1 (16.7) | 5 (27.8) | 0 | 0 | 0 | 0 |
| Fatigue | 1 (33.3) | 1 (10.0) | 0 | 2 (20.0) | 4 (11.8) | 2 (40.0) | 3 (42.9) | 3 (50.0) | 8 (44.4) | 10 (29.4) | 3 (75.0) | 10 (47.6) | 13 (52.0) |
| Headache | 0 | 1 (10.0) | 1 (9.1) | 2 (20.0) | 4 (11.8) | 0 | 0 | 0 | 0 | 0 | 0 | 0 | 0 |
| Hypothyroidism | 0 | 0 | 0 | 0 | 0 | 0 | 0 | 0 | 0 | 0 | 1 (25.0) | 5 (23.8) | 6 (24.0) |
| Infusion-related reaction | 0 | 0 | 0 | 0 | 0 | 0 | 0 | 0 | 0 | 10 (29.4) | 2 (50.0) | 5 (23.8) | 7 (28.0) |
| Nausea | 0 | 3 (30.0) | 2 (18.2) | 1 (10.0) | 6 (17.6) | 2 (40.0) | 0 | 4 (66.7) | 6 (33.3) | 6 (17.6) | 2 (50.0) | 11 (52.4) | 13 (52.0) |
| Neutrophil count decreased | 0 | 0 | 0 | 0 | 0 | 0 | 0 | 0 | 0 | 0 | 2 (50.0) | 7 (33.3) | 9 (36.0) |
| Oedema peripheral | 0 | 3 (30.0) | 1 (9.1) | 1 (10.0) | 5 (14.7) | 0 | 0 | 0 | 0 | 0 | 0 | 0 | 0 |
| Pyrexia | 0 | 0 | 0 | 0 | 0 | 2 (40.0) | 2 (28.6) | 1 (16.7) | 5 (27.8) | 0 | 0 | 0 | 0 |
| Rash maculo-papular | 0 | 0 | 0 | 0 | 0 | 2 (40.0) | 0 | 3 (50.0) | 5 (27.8) | 0 | 0 | 0 | 0 |
| Stomatitis | 0 | 0 | 0 | 0 | 0 | 0 | 0 | 0 | 0 | 0 | 1 (25.0) | 4 (19.0) | 5 (20.0) |
| Vomiting | 0 | 2 (20.0) | 1 (9.1) | 2 (20.0) | 5 (14.7) | 3 (60.0) | 1 (14.3) | 2 (33.3) | 6 (33.3) | 6 (17.6) | 2 (50.0) | 5 (23.8) | 7 (28.0) |

Bev, bevacizumab; dostar, dostarlimab; FOLFIRI, folinic acid, fluorouracil, irinotecan; mFOLFOX, modified folinic acid, 5-fluorouracil, oxaliplatin.

## Supplementary Table 5. Immune-related treatment-emergent adverse events

| **Preferred term, n (%)** | **Part 1A/B**  N=34 | | | | | **Part 1C**  N=18 | | | | **Part 2A** N=34 | **Part 2B** N=25 | | |
| --- | --- | --- | --- | --- | --- | --- | --- | --- | --- | --- | --- | --- | --- |
|  | **Encelimab 20 mg  (N=3)** | **Encelimab 80 mg   (N=10)** | **Encelimab 240 mg  (N=11)** | **Encelimab 720 mg  (N=10)** | **Total  (N=34)** | **Encelimab 80 mg + dostar**  **500 mg**  **(N=5)** | **Encelimab 240 mg + dostar**  **500 mg**  **(N=7)** | **Encelimab 720 mg + dostar**  **500 mg**  **(N=6)** | **Total (N=18)** | **Encelimab 720 mg + dostar 1000 mg (N=34)** | **Encelimab 720 mg + dostar 1000 mg + bev + mFOLFOX6 (N=4)** | **Encelimab 720 mg + dostar 1000 mg + bev + FOLFIRI (N=21)** | **Total (N=25)** |
| Alanine aminotransferase increased | 0 | 1 (10.0) | 0 | 0 | 1 (2.9) | 0 | 0 | 1 (16.7) | 1 (5.6) | 1 (2.9) | 1 (25.0) | 2 (9.5) | 3 (12.0) |
| Arthralgia | 1 (33.3) | 0 | 0 | 2 (20.0) | 3 (8.8) | 1 (20.0) | 1 (14.3) | 2 (33.3) | 4 (22.2) | 1 (2.9) | 0 | 0 | 0 |
| Aspartate aminotransferase increased | 0 | 1 (10.0) | 0 | 0 | 1 (2.9) | 1 (20.0) | 0 | 1 (16.7) | 2 (11.1) | 1 (2.9) | 1 (25.0) | 2 (9.5) | 3 (12.0) |
| Colitis | 0 | 0 | 0 | 0 | 0 | 0 | 0 | 0 | 0 | 0 | 0 | 1 (4.8) | 1 (4.0) |
| Encephalitis | 0 | 0 | 0 | 0 | 0 | 0 | 0 | 0 | 0 | 0 | 0 | 1 (4.8) | 1 (4.0) |
| Enteritis | 0 | 0 | 0 | 0 | 0 | 0 | 0 | 1 (16.7) | 1 (5.6) | 0 | 0 | 0 | 0 |
| Hyperthyroidism | 0 | 0 | 0 | 0 | 0 | 0 | 0 | 0 | 0 | 1 (2.9) | 0 | 0 | 0 |
| Hypothyroidism | 0 | 0 | 0 | 0 | 0 | 0 | 0 | 0 | 0 | 2 (5.9) | 1 (25.0) | 4 (19.0) | 5 (20.0) |
| Immune-mediated hepatitis | 0 | 0 | 0 | 0 | 0 | 0 | 0 | 1 (16.7) | 1 (5.6) | 0 | 0 | 1 (4.8) | 1 (4.0) |
| Infusion-related reaction | 0 | 1 (10.0) | 0 | 1 (10.0) | 2 (5.9) | 0 | 1 (14.3) | 2 (33.3) | 3 (16.7) | 10 (29.4) | 2 (50.0) | 3 (14.3) | 5 (20.0) |
| Myasthenia gravis | 0 | 1 (10.0) | 0 | 0 | 1 (2.9) | 0 | 0 | 0 | 0 | 0 | 0 | 0 | 0 |
| Pancreatitis | 0 | 0 | 0 | 0 | 0 | 0 | 1 (14.3) | 0 | 1 (5.6) | 0 | 0 | 0 | 0 |
| Pneumonitis | 0 | 0 | 0 | 0 | 0 | 0 | 0 | 0 | 0 | 1 (2.9) | 1 (25.0) | 0 | 1 (4.0) |
| Pruritus | 0 | 0 | 0 | 0 | 0 | 0 | 0 | 1 (16.7) | 1 (5.6) | 1 (2.9) | 0 | 0 | 0 |
| Rash | 0 | 0 | 0 | 0 | 0 | 1 (20.0) | 0 | 1 (16.7) | 2 (11.1) | 1 (2.9) | 0 | 1 (4.8) | 1 (4.0) |

Immune-related adverse events of interest were defined as any ≥Grade 2 AEs based on a prespecified search list of preferred terms.

AE, adverse event; bev, bevacizumab; dostar, dostarlimab; FOLFIRI, folinic acid, fluorouracil, irinotecan; mFOLFOX, modified folinic acid, 5-fluorouracil, oxaliplatin.

**Supplementary Table 6.** Tumour response summary per RECIST v1.1 for part 2 by PD-L1 and LAG-3 status

|  | **Part 2A**  **Encelimab 720 mg + dostar 1000 mg**  N=34 | | | | **Part 2B**  **Encelimab 720 mg +  dostar 1000 mg + bev + mFOLFOX6/FOLFIRI**  N=24 | | | |
| --- | --- | --- | --- | --- | --- | --- | --- | --- |
|  | **PD-L1 status** | | **LAG-3 status** | | **PD-L1 status** | | **LAG-3 status** | |
| **Biomarker status, n (%)** | | | | | | | | |
| Available | 22 (64.7) | | 18 (52.9) | | 16 (66.7) | | 15 (62.5) | |
| Missing | 12 (35.3) | | 16 (47.1) | | 8 (33.3) | | 9 (37.5) | |
| **Response measure by biomarker status** | **Positive**  N=12 | **Negative**  N=10 | **Positive**  N=13 | **Negative**  N=5 | **Positive**  N=6 | **Negative**  N=10 | **Positive**  N=14 | **Negative**  N=1 |
| **Best overall response, n (%)*** | | | | | | | | |
| CR | 0 | 0 | 0 | 0 | 0 | 0 | 0 | 0 |
| PR | 1 (8.3) | 0 | 1 (7.7) | 0 | 0 | 2 (20.0) | 2 (14.3) | 0 |
| SD | 1 (8.3) | 0 | 1 (7.7) | 0 | 4 (66.7) | 6 (60.0) | 8 (57.1) | 1 (100.0) |
| PD | 8 (66.7) | 10 (100.0) | 10 (76.9) | 5 (100.0) | 2 (33.3) | 2 (20.0) | 4 (28.6) | 0 |
| Not evaluable | 1 (8.3) | 0 | 0 | 0 | 0 | 0 | 0 | 0 |
| Not done | 1 (8.3) | 0 | 1 (7.7) | 0 | 0 | 0 | 0 | 0 |
| **Total** | **12 (100.0)** | **10 (100.0)** | **13 (100.0)** | **5 (100.0)** | **6 (100.0)** | **10 (100.0)** | **14 (100.0)** | **1 (100.0)** |

*Percentage values are calculated from the population with available biomarker status.

Bev, bevacizumab; CR, complete response; dostar, dostarlimab; FOLFIRI, folinic acid, fluorouracil, irinotecan; LAG-3, lymphocyte activation gene-3; mFOLFOX, modified folinic acid, 5-fluorouracil, oxaliplatin; ORR, objective response rate; PD, progressive disease; PD-L1, programmed death ligand-1; PR, partial response; RECIST, response evaluation criteria in solid tumours; SD, stable disease.

## Supplementary Figure 1. Study design


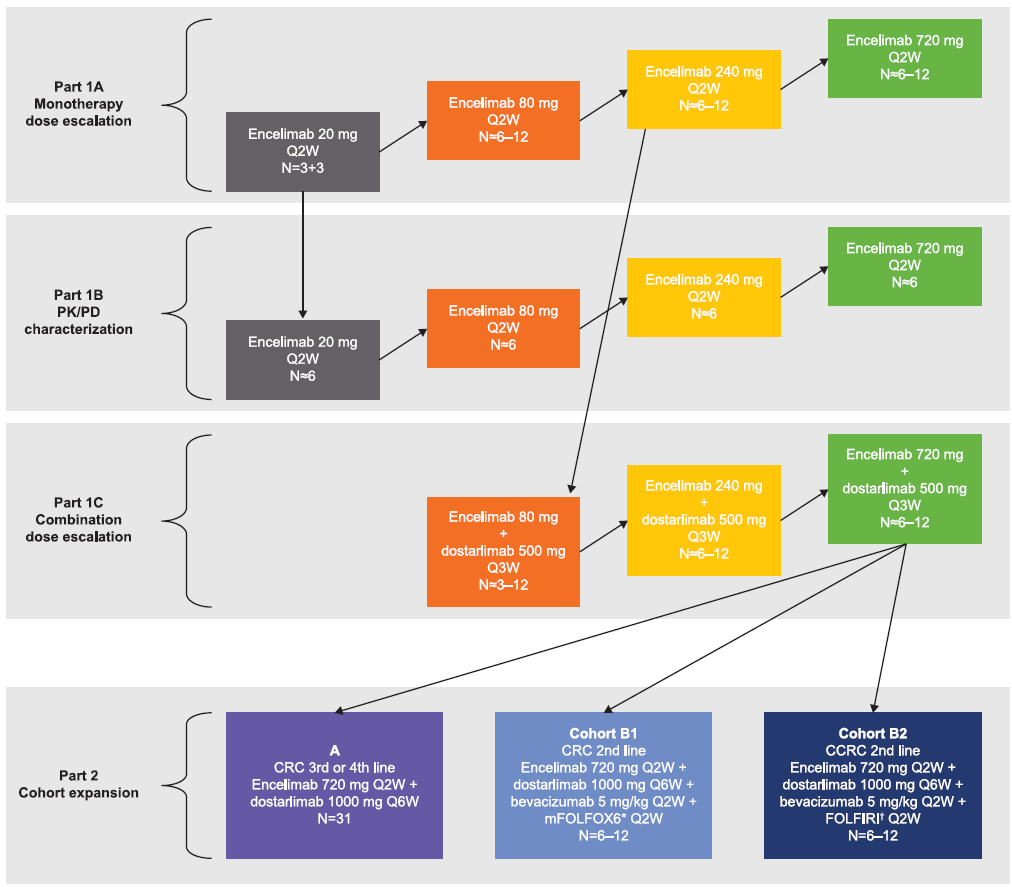


*mFOLFOX6 was administered as oxaliplatin 85 mg/m^2^ IV over 2 hours, leucovorin 400 mg/m^2^ IV over 2 hours, followed by 5-fluorouracil 400 mg/m^2^ IV bolus then continuous infusion of 5-fluorouracil 2400 mg/m^2^ over 46 hours. ^†^FOLFIRI was administered as irinotecan 180 mg/m^2^ IV over 90 minutes, leucovorin 400 mg/m^2^ IV over 90 minutes, followed by 5-fluorouracil 400 mg/m^2^ IV bolus then continuous infusion of 5-fluorouracil 2400 mg/m^2^ over 46 hours. Variations to schedules could be considered upon discussion with the medical monitor.

FOLFIRI, folinic acid, fluorouracil, irinotecan; IV, intravenous; mFOLFOX, modified folinic acid, 5-fluorouracil, oxaliplatin; PD, pharmacodynamic; PK, pharmacokinetic; Q*x*W, once every *x* weeks.

## Supplementary Figure 2. MRI tumour scans from responder patient at baseline (A), after 1 year on study treatment (B), and post study treatment discontinuation (C)


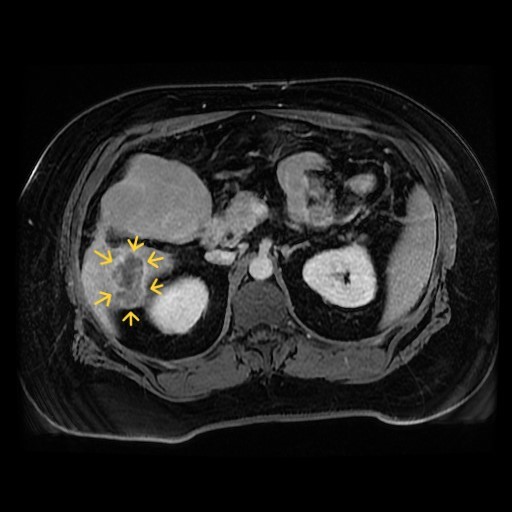


A


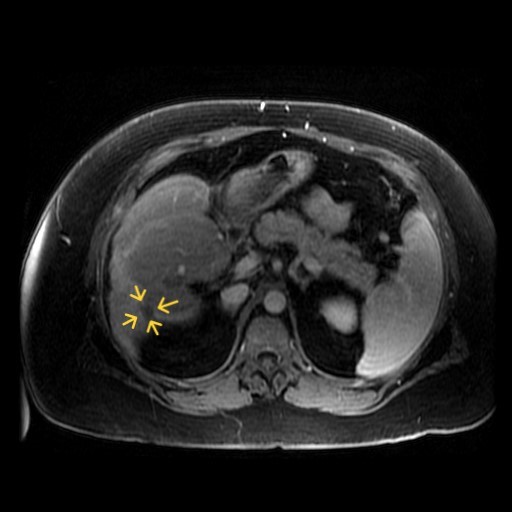


B


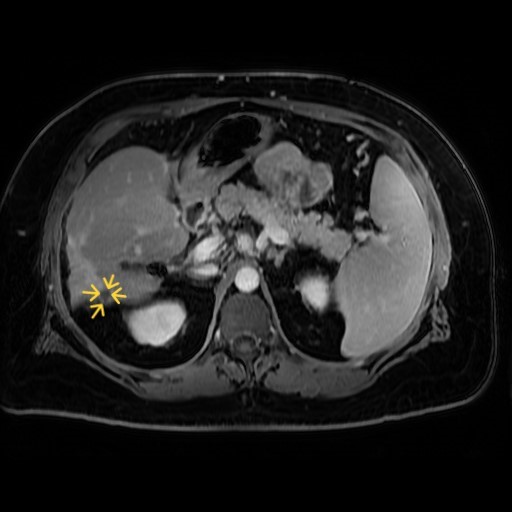


C

MRI, magnetic resonance imaging.

## Supplementary Figure 3. Mean serum concentration-time profile of encelimab dose 1 in part 1C (encelimab plus dostarlimab)


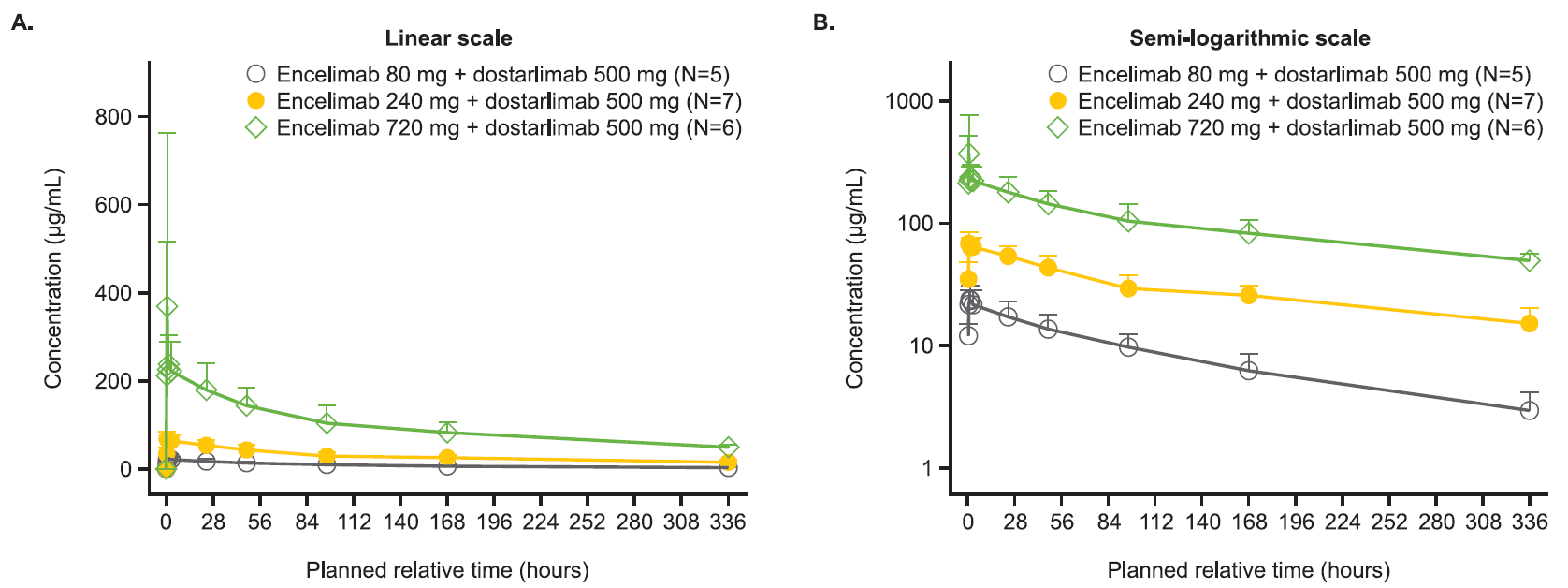


Data points show mean ± standard deviation.

## Supplementary Figure 4. LAG-3 receptor occupancy in part 1C (A and B; encelimab plus dostarlimab), part 2A (C; encelimab plus dostarlimab), and part 2B (D; encelimab plus dostarlimab plus bevacizumab plus mFOLFOX/FOLFIRI)

**
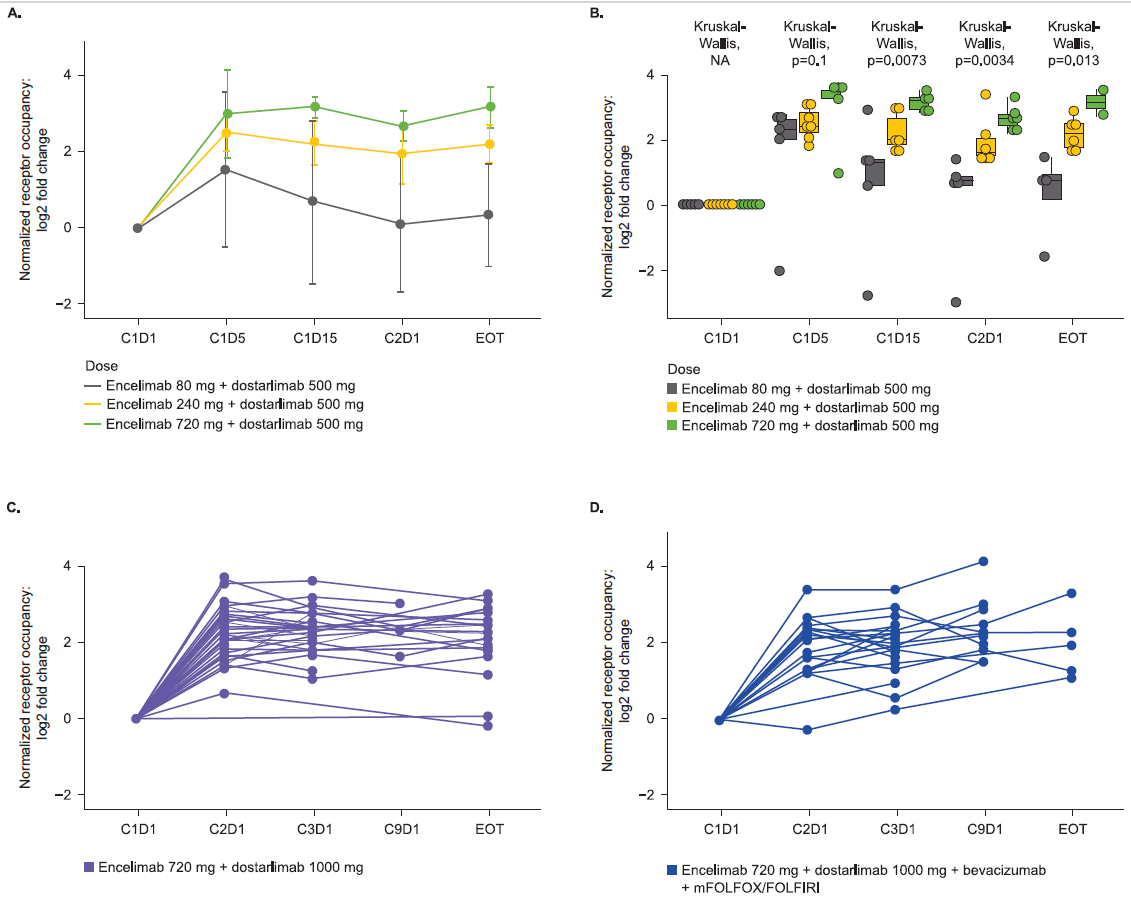
**

C, cycle; D, day; EOT, end of treatment; FOLFIRI, folinic acid, fluorouracil, irinotecan; LAG-3, lymphocyte activation gene-3; mFOLFOX, modified folinic acid, 5-fluorouracil, oxaliplatin; NA, not applicable.
